# The Buffer Effect of Social Identity on Psychological Stress in Different Competition Conditions

**DOI:** 10.3390/bs16030352

**Published:** 2026-03-02

**Authors:** Xiaohan Li, Kun Shi, Hua Zhang

**Affiliations:** 1 College of Laboratory Medicine, Chongqing Medical University, Chongqing 400715, China; 2 Faculty of Psychology, Southwest University, Chongqing 400715, China; qxz971024@email.swu.edu.cn; 3 Key Laboratory of Cognition and Personality, Ministry of Education, Southwest University, Chongqing 400715, China

**Keywords:** identity salience, stress reactivity, competition framing, psychophysiology

## Abstract

Psychological stress arises when perceived situational demands exceed an individual’s available coping resources. Beyond individual differences and broader contextual factors, an individual’s emotional connection to a group (i.e., social identity) may shape stress appraisals and physiological reactivity. Across two laboratory experiments with 180 college students, we examined whether making social identity salient influences acute stress responses under different competitive frames, comparing intragroup versus intergroup competition. In Experiment 1, participants in the social-identity condition showed numerically lower cardiovascular reactivity than those in the personal-identity condition, but between-condition differences were not statistically significant. In Experiment 2, the Identity × Competition interaction was statistically significant for heart-rate (HR) reactivity, indicating that the effect of identity salience differed across competition frames; however, this interaction did not generalize to systolic/diastolic blood pressure (SBP/DBP) or subjective stress. We also observed higher HR reactivity in intragroup than intergroup competition in this protocol, which we interpret cautiously given the limited consistency across outcomes. Overall, the findings suggest that any identity-related modulation of acute stress responding may be context-dependent and modality-specific, underscoring the importance of competitive framing when evaluating the stress-related consequences of social identity.

## 1. Introduction

Stress is an adaptive response mobilized when physiological or psychological homeostasis is challenged (Levine, 2005). It involves coordinated psychological and physiological changes (Taverniers et al., 2010), including increased anxiety (Rimmele et al., 2007), reduced subjective calm (Childs & Stoeber, 2012), and heightened negative affect (Firk & Markus, 2009). Although acute stress can be functional, chronic or excessive stress taxes coping resources and is linked to serious disorders and elevated mental health risks (Wills & Langner, 1980; Juster et al., 2011; Stout & Nemeroff, 1994). Cognitive appraisal theory emphasizes that stress arises from person–environment evaluations (Lazarus & Folkman, 1984), including primary appraisals (threat vs. challenge) and secondary appraisals (available coping resources; Lazarus & Folkman, 1984). Importantly, appraisals are subjective yet socially embedded (Haslam, 2004). For example, situational appraisals explain meaningful variance in TSST cortisol responding, highlighting the role of context over and above trait factors (Gaab et al., 2005). Thus, identifying situational determinants that shift threat–challenge appraisals remain central to understanding stress reactivity.

Stress responding varies across contexts, partly because self-definition is context-sensitive and depends on which identity is salient (Haslam & Reicher, 2006). Social identity theory proposes that the self is multi-level (personal vs. social) and that salience determines which self-representation guides appraisal and perceived coping resources in a given situation (Tajfel & Turner, 1979). Personal identity (the “I”) reflects idiosyncratic attributes, whereas social identity (the “we”) is derived from ingroup membership and its norms (Tajfel & Turner, 1979). When group membership becomes salient, individuals construe group goals, norms, and outcomes as self-relevant, shaping behavior across schools, workplaces, and broader collectives (van Dick et al., 2017). This salience shift is therefore a plausible route through which identity can alter stress appraisals.

When people self-categorize as group members, primary appraisals are referenced to ingroup norms and identity relevance (Reicher & Levine, 1994), and secondary appraisals may draw on perceived collective efficacy, belonging, and shared resources (Gallagher et al., 2014). On this basis, salient group membership can buffer stress responses—yet under some conditions, it may amplify arousal by increasing the evaluative stakes attached to performance and self-worth (Haslam, 2004). Consistent with a “social cure” account, many studies report reduced stress when a shared identity is salient (e.g., Häusser et al., 2012; Frisch et al., 2014; Hopkins et al., 2015; Stevenson, 2016), and recent work continues to emphasize that identification and group membership are linked to more adaptive appraisal profiles and stress-related outcomes (Gillman et al., 2023; McMahon et al., 2024). However, other findings indicate null effects or heightened stress under stronger identification or identity salience (Kuppens & Yzerbyt, 2012; Isaksson et al., 2017; Erkens et al., 2018), and recent experimental evidence likewise shows that making a stigmatized or stereotype-relevant identity salient can amplify blood pressure responses under stress (Skilton et al., 2025). These inconsistencies suggest that identity’s role in stress is context-dependent, potentially shifting from resource-enhancing to demand-amplifying.

Competition is a particularly relevant context because it entails negatively interdependent outcomes: one person’s success implies others’ failure (Deutsch, 2016). As a normative social comparison structure, competition yields clear winners and losers (Murayama & Elliot, 2012). In intergroup competition, comparisons are drawn between groups and often foster antagonism and outgroup devaluation (Rabbie & Wilkens, 1971), while simultaneously aligning members around a shared group goal that can increase cohesion (Boulding, 1962). Intragroup competition instead emphasizes within-group comparison, rank, and relative standing. Both forms can function as acute stressors, elevating cardiovascular responding (e.g., Harkins et al., 1992; McKay et al., 1997; Harrison et al., 2010; Wittchen et al., 2013), but they are likely to engage different appraisal pathways—making competition framing a plausible boundary condition for identity-based buffering.

A robust phenomenon in intergroup relations is the interindividual–intergroup discontinuity effect: individuals often behave more competitively and aggressively in intergroup than interindividual contexts (Insko et al., 1998). Explanations of this discontinuity typically invoke social identity processes, arguing that the effect is stronger when identity salience, group norms, and status concerns are foregrounded (Böhm et al., 2013). In the present research, we therefore treat competition framing (intragroup vs. intergroup) as a contextual moderator that may determine when identity salience functions as a buffer versus a liability in stress responding.

From a social identity perspective, self-categorization triggers social comparison and motives the maintenance of positive ingroup distinctiveness (Tajfel, 1986). Intergroup competition intensifies such comparisons and elevates identity relevance (Hamilton et al., 1998), strengthening motives to protect ingroup standing and differentiate from outgroups (Tajfel, 1986). When the ingroup’s standing is (or could be) devalued, people may experience social identity threat (SIT)—the concern that self-worth will be undermined due to group membership (e.g., Isaksson et al., 2017). Notably, intergroup comparison itself can elicit threat-relevant appraisals (Stephan et al., 2000; Q. Zhang et al., 2009), and identity-threatening stressors have been linked to divergent physiological outcomes, including blood pressure elevations, depending on appraisal processes (e.g., Reeves et al., 2024). Accordingly, we expect identity salience to buffer stress in intragroup contexts (where shared identity may increase perceived resources) but to be attenuated under intergroup framing, where threat-related appraisals may counteract buffering by increasing perceived demands.

The growing literature supports the idea that SIT can heighten stress responses when identity-relevant cues are present. For example, individuals with strong ingroup identification show elevated blood pressure in identity-relevant competitive contexts (Branscombe & Wann, 1992), people facing devalued identities report greater stress when confronting identity-congruent evaluative tasks (Isaksson et al., 2017), and identity-salience manipulations can amplify cardiovascular reactivity under stereotype-relevant stressors (Skilton et al., 2025). Together, these findings imply that when identity salience increases the stakes for self-worth maintenance, SIT may counteract any buffering and lead to greater stress reactivity under threat-laden competitive contexts.

Despite extensive work on social identity and stress, findings remain mixed, suggesting that identity can function as a resource in some contexts but become a source of demand in others. The present research addresses this inconsistency in two ways. First, we test whether identity salience per se—without directly providing social support—shifts stress appraisals and reduces acute psychophysiological reactivity. Second, we examine whether competition framing (intragroup vs. intergroup) serves as a key boundary condition by altering the likelihood of SIT-related threat appraisals, thereby helping reconcile prior discrepant findings. This focus aligns with recent syntheses linking social identity to health-relevant outcomes while emphasizing the importance of specifying when identity is beneficial versus costly (de Hoog & Pat-El, 2024).

Guided by this framework, we ask whether making social identity salient (vs. personal identity) reduces acute stress reactivity under a standardized laboratory stressor and whether this effect depends on competition framing—emerging more reliably in intragroup contexts but weakening under intergroup framing where identity stakes may increase perceived demands. To test these questions, we conducted two laboratory experiments using the PASAT and multimethod assessment (HR, SBP, DBP, and self-reported stress). Experiment 1 isolated the main effect of identity salience (social vs. personal). Experiment 2 orthogonally manipulated identity salience and competition type to test the predicted moderation.

## 2. Experiment 1

Experiment 1 used a laboratory stress paradigm to test whether making a social identity salient—without providing direct social support—reduces acute stress reactivity relative to a personal-identity salience condition. Stress responses were indexed using cardiovascular measures (heart rate and systolic/diastolic blood pressure) and a self-reported stress rating during a standardized cognitive stressor (PASAT). We adopted a pre–post design and quantified reactivity as the change from baseline to task.

### 2.1. Methods

#### 2.1.1. Participants

We recruited 60 undergraduates; 2 were excluded due to ECG attachment problems, yielding a final sample of *N* = 58 (35 women and 23 men; *M*age = 20.26 and *SD* = 1.52). Inclusion criteria were normal vision/hearing and no self-reported cardiovascular, neurological, or psychiatric disorders. Participants were randomly assigned (1:1) to the social-identity versus personal-identity salience conditions and received 15 RMB upon completion.

Sensitivity analysis: Given the final sample size, we report a sensitivity analysis to clarify the minimum effect size that can be detected with adequate power. A sensitivity analysis (G*Power 3.1; α = 0.05 and 1 − β = 0.80; two-tailed independent-samples comparison with equal group sizes) indicated that with N = 58, the minimum detectable between-group effect is approximately Cohen’s d = 0.75 (equivalently, Cohen’s f = 0.37 and η^2^ ≈ 0.12). Accordingly, effects smaller than this threshold may not be reliably detected in Experiment 1, and non-significant results should be interpreted as inconclusive rather than confirmatory evidence for the null. This minimum effect estimate is reported as a transparent sensitivity benchmark for the primary between-condition effect in Experiment 1; because the main analysis used ANCOVA (with covariates), the estimate should be interpreted as an approximate reference rather than an exact power calculation for the covariate-adjusted model.

#### 2.1.2. Experimental Material

Baseline social identity (Hogg & Turner, 1987): Eight items were placed on a 7-point Likert scale (1 = not at all and 7 = very much); sample items include “I identify as a member of Southwest University” and “I strongly identify with Southwest University.” Internal consistency was high (α = 0.903).

Social Identity Operationalization Questionnaire (SIOQ; Luhtanen & Crocker, 1992; C. Zhang, 2011 translation): Four items were placed on a 7-point Likert scale; α = 0.814. Example items are “I identify as a worthy member of my university group” and “I often feel I’m not an important member of my group” (reverse-coded).

Identity-salience manipulation task (Three-Events Identity Task; Haslam et al., 1999): In the personal-identity condition, participants listed three features that distinguish them from others; in the social-identity condition, they listed three similarities or shared strengths with “Southwest University students.” This task served as the manipulation rather than an outcome measure. Two blind coders classified each entry as group-referent (1) or personal-referent (0). The mean across the three entries formed an identity-referent index; inter-coder agreement (κ) is reported in the Supplementary Materials.

Trait and state anxiety (STAI-Y; Spielberger, 1983): Trait anxiety was used as a covariate, and state anxiety was assessed as an affective correlate. Reported reliabilities are α = 0.88/0.90.

Subjective stress: A single-item stress rating (1–7) was administered post-task to avoid interrupting physiological recording; this timing is discussed as a limitation.

Physiology: ECG (Biopac MP150, 1000 Hz; three electrodes) was used to derive HR; an Omron HEM-1020 device was used to assess SBP/DBP.

#### 2.1.3. Task and Procedure

During online recruitment, participants completed demographic information, baseline social identity (Hogg & Turner, 1987), and STAI-Trait. On the laboratory day, participants arrived at Room A, were fitted with ECG sensors, and completed a 5 min seated resting baseline prior to any manipulation or task instructions.

After baseline, participants entered an anticipation/instruction phase during which the identity-salience manipulation was administered. In the social-identity condition, participants completed the social-identity Three-Events prompts, drew a Southwest University-related image, and wore a vest with the university logo. In the personal-identity condition, participants completed the personal-identity Three-Events prompts and drew a self-image. To align with physiological sampling, the anticipation phase was segmented into four 2 min windows (A1–A4). State anxiety was assessed after this phase to avoid interrupting the physiological windows.

Blood pressure (SBP/DBP) was assessed every 2 min during A1–A4 (T1–T4), alternating left/right arms; HR was averaged within the corresponding 2 min windows. Participants then moved to Room B for the PASAT (4 min). Following a brief practice/understanding check (~20 s), the formal task proceeded in two 2 min segments (P1 and P2). BP was measured at the end of each segment (T5, T6), and HR was averaged within P1 and P2. Immediately after PASAT, participants returned to Room A and completed the manipulation check, STAI-State, and the subjective stress rating and then were debriefed and compensated.

To minimize procedural disruption, measurements were scheduled at standardized time points and instructions were kept brief. Nonetheless, repeated cuff inflation and posture/arm adjustments may increase procedural salience and divert attention from experimental framing; we treat this as a potential source of attenuation of between-condition effects.

### 2.2. Results

#### 2.2.1. Preliminary Equivalence Checks

To assess pre-experimental equivalence, we conducted independent-samples tests for trait anxiety, state anxiety, and baseline social identity. No significant group differences emerged (Table S1 in the Supplementary Materials). Trait anxiety and baseline identity were retained as covariates in primary analyses to improve precision.

#### 2.2.2. Manipulation Check and Task Reactivity

To verify that PASAT elicited stress responses, we tested within-subject change from baseline to task for subjective stress and cardiovascular indices. The PASAT produced a significant time effect across measures, indicating reliable psychophysiological reactivity (Table S2 in the Supplementary Materials). For the identity-salience manipulation check, an ANCOVA controlling for baseline identity showed higher identity-salience scores in the social-identity condition than in the personal-identity condition, indicating successful manipulation (Table S3 in the Supplementary Materials).

#### 2.2.3. Effect of Identity Salience on Stress Reactivity

For each outcome, reactivity was computed as T0 = mean(P1–P2) − mean (Baseline). We then conducted one-way ANCOVAs predicting T0 based on the condition (social vs. personal), controlling for trait anxiety and baseline identity. Condition effects on HR, SBP, DBP, and subjective stress reactivity did not reach α = 0.05 (Table 1). We therefore interpret the evidence as inconclusive with respect to a confirmatory buffering effect in Experiment 1. We assessed key assumptions for the ANOVA/ANCOVA and repeated-measures models. Specifically, we inspected residual distributions, tested homogeneity of variance using Levene’s tests, evaluated homogeneity of regression slopes in ANCOVA by including covariate × factor interaction terms, and examined sphericity for repeated-measures analyses using Mauchly’s test with Greenhouse. These diagnostics did not indicate violations that would materially affect inference, and the pattern of results remained unchanged under the appropriate corrections.

### 2.3. Discussion

Experiment 1 examined whether making social identity salient—without directly providing social support—reduces acute stress reactivity relative to personal-identity salience. Across HR, SBP, and DBP, the social-identity condition showed numerically smaller reactivity than the personal-identity condition; however, the between-condition effects did not reach statistical significance. Consistent with current inferential standards, we therefore treat these findings as inconclusive rather than confirmatory evidence for identity-based buffering.

The PASAT reliably elicited stress responses, yet the absence of robust between-condition differences may reflect both measurement constraints and the known partial dissociation between physiological reactivity and retrospective self-reports in acute stress paradigms. In particular, the subjective stress rating was assessed post-task to avoid interrupting the protocol; such end-point ratings can be influenced by recall and immediate relief and may not capture peak activation during the task (Lazarus & Folkman, 1984). In addition, repeated cuff-based blood pressure assessment (including posture/arm adjustments) can increase procedural salience and introduce variance unrelated to the manipulation, potentially attenuating subtle condition effects—an issue noted in psychophysiological measurement contexts where task demands and recording procedures compete for attention (Häusser et al., 2012; Gaab et al., 2005).

Importantly, null or weak effects in Experiment 1 do not preclude the possibility that identity salience matters under specific contextual frames. Social identity theory and related work suggest that identity can function as a resource when it increases perceived collective efficacy but can become costly when identity-relevant evaluation heightens threat appraisals (Haslam & Reicher, 2006; Tajfel & Turner, 1979). This contextual sensitivity offers a plausible account for mixed findings in the literature (Häusser et al., 2012; Isaksson et al., 2017). Accordingly, Experiment 2 tests whether competition framing (intragroup vs. intergroup) moderates the effect of identity salience, such that identity is more likely to buffer reactivity in intragroup contexts but be attenuated under intergroup framing where identity stakes and social identity threat may increase (Blascovich et al., 2001; Isaksson et al., 2017).

## 3. Experiment 2

Experiment 2 used the same laboratory stress paradigm and identity-salience manipulation as Experiment 1 and additionally orthogonally manipulated competition framing (intragroup vs. intergroup). The aim was to test whether the effect of identity salience on acute stress reactivity is moderated by competitive context.

### 3.1. Methods

#### 3.1.1. Participants

We recruited 120 undergraduates; 1 was excluded due to ECG attachment problems, yielding N = 119 (72 women and 47 men; *M*age = 19.95 and *SD* = 1.86). Inclusion criteria were normal vision/hearing and no self-reported cardiovascular, neurological, or psychiatric disorders. Participants were randomly assigned to a 2 (Identity: social vs. personal) × 2 (Competition: intragroup vs. intergroup) between-subjects design with approximately equal cell sizes. Each participant received 15 RMB upon completion.

#### 3.1.2. Materials and Measures

Materials and measures were identical to Experiment 1 (baseline social identity, SIOQ for identity salience, Three-Events task, STAI-Y Trait/State, single-item subjective stress, ECG-derived HR, Omron-based SBP/DBP, and vests). In addition, competition framing was manipulated via a situational scenario:

Intergroup competition (between universities): Instructions emphasized that Southwest University was competing against Chongqing University and Chongqing Normal University, that participants’ accuracy contributed to university ranking, and that subsequent funding would be tied to performance (scenario text retained in materials).

Intragroup competition (within university): Instructions emphasized ranking among Southwest University students, advancement for the top 20%, and within-group comparison (scenario text retained in materials).

A competition manipulation check (“I felt a competitive atmosphere…”, 0–9 slider) assessed perceived competitiveness at the end of the task.

#### 3.1.3. Task and Procedure

The procedure matched Experiment 1 with the following addition: after the 5 min baseline (B), participants received competition framing instructions and completed the identity-salience manipulation during an anticipation/instruction phase segmented into four 2 min windows (A1–A4), aligned with BP (end of each window; arms alternating) and HR (window means). Participants then completed the PASAT (4 min) in two 2 min segments (P1–P2) with BP measured at T5/T6 and HR averaged within P1 and P2. Post-task, participants completed the manipulation checks, STAI-State, and the 1–7 subjective stress rating and then were debriefed and compensated.

### 3.2. Results

#### 3.2.1. Preliminary Equivalence Checks

We examined pre-experimental equivalence across conditions for baseline social identity, state anxiety, and trait anxiety. Baseline identity and state anxiety did not differ across groups (Table S4 in the Supplementary Materials).

#### 3.2.2. Task Reactivity and Manipulation Checks

Repeated-measures analyses showed a significant time effect for subjective stress and cardiovascular indices, confirming that PASAT elicited psychophysiological reactivity (Table S5 in the Supplementary Materials). For the identity-salience manipulation check, ANCOVA controlling baseline identity indicated higher identity-salience scores in the social-identity condition than in the personal-identity condition (Table S6). The competition framing manipulation check indicated that perceived competitiveness differed between competition conditions (Table S6 in the Supplementary Materials).

#### 3.2.3. Identity Salience Across Competitive Contexts

Reactivity was computed as T0 = mean(P1–P2) − mean (Baseline). We then conducted 2 (Identity: social vs. personal) × 2 (Competition: intragroup vs. intergroup) ANCOVAs on T0 for HR, SBP, DBP, and subjective stress, with trait anxiety as a covariate.

**Figure 1:**
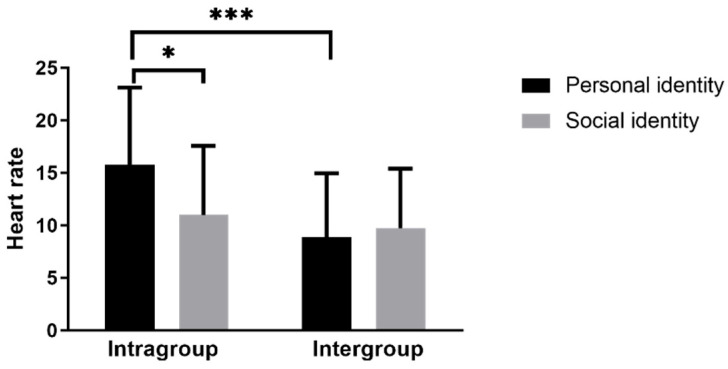
Experiment 2: heart rate reactivity (T0). Note. (* *p* < 0.05 and *** *p* < 0.001).

For HR reactivity, there was a significant main effect of Competition: *F* (1, 114) = 14.97, *p* < 0.001, and *η_p_*^2^ = 0.116. Importantly, the Identity × Competition interaction was also significant, *F* (1, 114) = 5.28, *p* = 0.023, and *η_p_*^2^ = 0.044, indicating that the effect of identity salience differed across competition frames. To characterize this interaction, we examined simple effects: under intragroup competition, the social-identity condition showed lower HR reactivity than the personal-identity condition, whereas under intergroup competition, the two identity conditions did not differ (Table 2; Figure 1).

For SBP reactivity, the main effect of Competition was significant: *F* (1, 114) = 4.65, *p* = 0.033, and *η_p_*^2^ = 0.039. DBP and subjective stress showed no statistically reliable main effects or interactions (Table 2).

Model assumptions (residual diagnostics, Levene’s tests, and homogeneity of regression slopes) were examined for all primary ANCOVA models; no violations were detected that would materially affect inference.

### 3.3. Discussion

Experiment 2 tested whether competition framing moderates the effect of identity salience on acute stress reactivity. The PASAT elicited robust reactivity. For HR reactivity, the predicted Identity × Competition interaction was significant, indicating that identity salience operated differently across competitive contexts. Specifically, social-identity salience was associated with lower HR reactivity under intragroup competition, whereas no identity difference emerged under intergroup competition (Table 2; Figure 1). Although the interaction effect was small-to-moderate (*η_p_*^2^ = 0.044) and did not generalize to SBP/DBP or subjective stress, the HR pattern is consistent with a context-dependent account of identity-related stress responding.

Within the biopsychosocial challenge–threat framework, intergroup framing may increase identity-relevant stakes and perceived demands, shifting appraisals toward threat and counteracting resource-based benefits of identity salience (Blascovich et al., 2001; Mendes et al., 2002). This explanation also aligns with social identity threat perspectives linking identity-relevant evaluation to heightened arousal and performance concerns (Steele & Aronson, 1995; Isaksson et al., 2017). SBP reactivity also differed across competition frames, whereas DBP and retrospective subjective stress showed no reliable moderation effects. We emphasize that this challenge–threat/SIT account is inferential rather than directly tested in the present study, because we did not measure appraisals, perceived demands/resources, social identity threat, or hemodynamic indices (e.g., CO/TPR). Accordingly, this mechanism should be treated as a theoretically plausible interpretation and a hypothesis for future work.

Two issues qualify inference. Intergroup framing increased perceived competitiveness and may also elevate identity relevance, implying partial overlap between framing and salience in participants’ subjective experience. In addition, repeated cuff-based BP assessment may have increased procedural salience and introduced noise. Future work should use less intrusive continuous monitoring and include hemodynamic indicators (e.g., cardiac output and total peripheral resistance) to better distinguish challenge-related engagement from threat-related responding (Blascovich & Tomaka, 1996), ideally in more interactive intergroup settings.

## 4. General Discussion

Across two laboratory experiments using the PASAT, we examined whether making a social identity salient (vs. personal identity) modulates acute stress responding in the absence of enacted social support and whether any identity–stress association depends on competition framing (intragroup vs. intergroup). In both experiments, the PASAT reliably increased cardiovascular activation, supporting its suitability as an acute cognitive stressor. Evidence for a main-effect “buffering” account was limited: in Experiment 1, the social-identity condition showed numerically smaller cardiovascular reactivity than the personal-identity condition, but between-condition differences were not statistically significant. In Experiment 2, competition framing showed a robust main effect on heart-rate (HR) reactivity, and the Identity × Competition interaction was statistically significant for HR, indicating that identity salience operated differently across competition frames; however, this moderation did not generalize to SBP/DBP or subjective stress. Accordingly, the most defensible conclusion is that identity-related modulation in this protocol is small and measure-specific: we observed a statistically reliable Identity × Competition effect for HR but not for SBP/DBP or retrospective subjective stress.

### 4.1. Theoretical Implications: Identity as a Resource—And as a Demand

A core premise of the social identity approach is that shifts in self-definition (from “I” to “we”) can restructure appraisal by changing what is at stake and what resources are perceived as available (Haslam et al., 2009). Within transactional appraisal models, stress responses emerge from primary appraisal (threat/challenge significance) and secondary appraisal (coping resources) (Lazarus & Folkman, 1984). Social identity salience can plausibly increase perceived resources (e.g., collective efficacy, meaning, and shared standards), which should facilitate more benign appraisals and lower reactivity in some contexts (Haslam et al., 2009). At the same time, identity salience can increase perceived demands when performance becomes diagnostic of the group’s standing, thereby shifting appraisal toward threat. This dual-role logic is consistent with work showing that identity-related cues can either protect or amplify physiological stress depending on whether they imply evaluation or devaluation of the self as a group member (Skilton et al., 2025; Steele & Aronson, 1995).

The biopsychosocial model of challenge and threat provides a useful organizing lens for the present pattern because it formalizes stress responding as a demands–resources balance (Blascovich & Tomaka, 1996; Mendes et al., 2002). In this framework, intergroup competition can inflate perceived demands (e.g., reputational consequences for the ingroup; group-based evaluation), potentially overriding any resource benefits conferred by identity salience. Conversely, intragroup competition can elicit strong activation via effortful engagement without necessarily implying group devaluation, meaning that “more arousal” is not isomorphic with “more threat” unless hemodynamic patterns (e.g., CO/TPR) are assessed (Blascovich & Tomaka, 1996). This is one reason we avoid interpreting the competition main effect on HR as direct evidence of threat versus challenge.

### 4.2. Competition Framing and the Boundary Conditions of Identity-Based Buffering

Experiment 2 adds a boundary-condition test aligned with classic work on intergroup processes and competitive framing (Insko et al., 1998). In our data, competition framing produced a reliable shift in HR reactivity, indicating that the “frame” mattered psychologically. However, the predicted moderation by identity salience was statistically supported for HR reactivity, although the moderation pattern did not extend to SBP/DBP or subjective stress, suggesting that either (a) the true interaction is small, or (b) the present operationalization produced overlapping subjective experiences that reduced separability of identity and competition effects, or (c) measurement noise attenuated subtle between-condition differences.

Two theoretical points follow. First, although we observed a statistically significant Identity × Competition interaction for HR reactivity, the moderation pattern did not extend to SBP/DBP or subjective stress, suggesting that the boundary condition may be modality-specific and/or sensitive to measurement noise. Second, intergroup framing may not simply “remove” buffering; it can plausibly transform identity into an additional performance demand (identity-relevant evaluation), consistent with broader models of social-evaluative threat across levels of self and challenge–threat dynamics (Blascovich & Tomaka, 1996; Mendes et al., 2002). Recent applied work also supports this appraisal pathway: social identification is associated with more challenge-oriented appraisals in stressful work experiences, with downstream links to perceived stress and functioning (Gillman et al., 2023).

### 4.3. Methodological Contributions and Interpretation Discipline

This manuscript makes two methodological contributions that remain valuable even under largely inconclusive hypothesis tests. First, we isolated identity salience from enacted support, which helps clarify that “identity effects” in the stress literature are not reducible to direct comforting behavior (Haslam et al., 2009). Second, we implemented a phase-aligned psychophysiological protocol and transparent reactivity computation. At the same time, the present results underscore an interpretive discipline emphasized by reviewers: non-significant effects cannot be used to “support” hypotheses. Under the current design, the most appropriate reading is that the evidence is null/inconclusive for key identity effects, rather than confirmatory.

The divergence between cardiovascular indices and retrospective self-reports is also theoretically interpretable. Social-evaluative stress research shows that physiological reactivity can be temporally sharp and phase-specific, whereas end-point self-reports compress dynamics and can reflect post-task relief, performance impressions, and demand characteristics (Dickerson & Kemeny, 2004). This measurement timing issue is especially salient when subjective stress is assessed only after task completion, while physiology reflects moment-to-moment responding during anticipation and performance (Gaab et al., 2005). Thus, null self-report differences here should not be taken as evidence that participants “did not feel stress,” but rather that the self-report window may not have been optimally sensitive to condition differences.

### 4.4. Practical Implications and Limitations

The present findings do not justify a blanket claim that identity salience buffers stress. Rather, they suggest that identity cues are most likely to be useful when they increase perceived resources (e.g., belonging, shared standards, and collective efficacy) without simultaneously intensifying identity-relevant evaluative stakes. In applied settings such as classrooms, teams, or organizational units, this implies that practitioners should emphasize shared goals and ingroup norms while avoiding framings that make performance strongly diagnostic of group worth or public intergroup status comparison—conditions that are more likely to shift appraisals toward threat (Haslam et al., 2009; Lazarus & Folkman, 1984; Blascovich & Tomaka, 1996).

The present findings should be interpreted within several design boundaries. Experiment 1 had limited sensitivity to detect small psychophysiological effects, so its null findings are better treated as inconclusive rather than as evidence of no effect. In addition, repeated cuff-based blood pressure assessment (including arm switching) may have increased procedural salience and introduced task-irrelevant variance, potentially attenuating subtle condition differences (Dickerson & Kemeny, 2004). The identity-salience manipulation also combined multiple components (writing, drawing, and apparel), which reduces construct specificity regarding which element contributed most to the observed pattern. At the same time, the use of a single-university undergraduate sample and scenario-based competition framing, while advantageous for experimental control, limits the extent to which the findings can be generalized to other populations and to ecologically richer competitive settings.

These considerations point to a clear next step for the field: higher-powered studies using less intrusive physiological recording and more diagnostic indicators of challenge–threat processes—especially hemodynamic markers such as cardiac output and total peripheral resistance—so that effortful engagement can be distinguished more directly from threat-related responding (Blascovich & Tomaka, 1996). It will also be important to test the proposed boundary conditions in more diverse samples and in more interactive or field-based competitive contexts.

## 5. Conclusions

In summary, across two experiments without direct social support, making social identity salient tended to relate to lower cardiovascular reactivity, with context qualifying this pattern: intragroup framing was more compatible with buffering, whereas intergroup framing may introduce identity-threat appraisals that attenuate benefits. Some indicators suggested higher reactivity under intragroup than intergroup competition in this setting; given mixed significance levels, we interpret these differences cautiously and highlight the need for richer measurement and interactive paradigms in future work.

## Figures and Tables

**Table 1:** Baseline vs. task (time effect) for physiological data in Experiment 1.

		Social Identity (*N* = 29) *M* (*SD*)	Personal Identity (*N* = 29) *M* (*SD*)	*F*	*η_p_* ^2^
Heart rate	Baseline period	78.48 ± 10.02	79.65 ± 6.20	6.18 *	0.101
	Task period (PASAT)	86.79 ± 12.07	92.80 ± 12.18		
	T0	8.49 ± 6.69	13.37 ± 8.01		
Systolic blood pressure	Baseline period	96.84 ± 8.92	98.26 ± 13.44	4.36 *	0.074
	Mandate period	102.29 ± 10.29	107.29 ± 12.90		
	T0	5.67 ± 6.16	9.39 ± 6.72		
Diastolic blood pressure	Baseline period	58.39 ± 7.64	56.91 ± 10.28	6.92 *	0.112
	Task period (PASAT)	63.94 ± 6.41	66.31 ± 11.19		
	T0	5.92 ± 5.38	9.89 ± 6.39		
Subjective stress report	Baseline period	3.52 ± 1.40	3.19 ± 1.44	0.74	0.013
	Task period (PASAT)	4.62 ± 1.08	4.97 ± 1.40		
	T0	1.10 ± 1.76	1.69 ± 2.65		

Note: (1) T0 = mean(P1–P2) − mean(B). Analyses are ANCOVAs controlling for trait anxiety and baseline identity. Values are M (SD). (2) * *p* < 0.05.

**Table 2:** ANCOVA results for reactivity (T0) outcomes in Experiment 2.

	Type III Sum of Squares	*df*	Dependent Variable	*F*	*η_p_* ^2^
Trait anxiety	180.04	1	Systolic blood pressure T0	0.21	0.002
	19.67	1	Diastolic blood pressure T0	0.42	0.004
	10.61	1	Heart rate T0	4.43 *	0.037
Identity	72.22	1	Systolic blood pressure T0	0.70	0.006
	67.13	1	Diastolic blood pressure T0	1.45	0.013
	36.10	1	Heart rate T0	1.78	0.015
Competition	607.83	1	Systolic blood pressure T0	4.65 *	0.039
	0.39	1	Diastolic blood pressure T0	0.01	0.000
	238.91	1	Heart rate T0	14.97 ***	0.116
Identity * Competition	214.29	1	Systolic blood pressure T0	0.16	0.001
	4.81	1	Diastolic blood pressure T0	0.10	0.001
	8.02	1	Heart rate T0	5.28 *	0.044

Note. T0 = mean(P1–P2) − mean (Baseline). Table reports Type III sums of squares, *df*, *F*, and partial *η*^2^ from 2 × 2 ANCOVAs with trait anxiety as a covariate. *p* values correspond to the F-tests (* *p* < 0.05, and *** *p* < 0.001).

## Data Availability

All data and materials are available. Please contact the author to request them.

## Supplementary Materials

The following supporting information can be downloaded at: https://www.mdpi.com/article/10.3390/bs16030352/s1. Table S1: STAI and baseline level of identity data in Experiment 1; Table S2: Psychogenic Stress Validity Test in Experiment 1; Table S3: Social identity highlights validity tests in Experiment 1; Table S4: STAI and baseline level of identity data in Experiment 2; Table S5: Psychogenic Stress Validity Test in Experiment 2; Table S6: Social identity highlights validity tests in Experiment 2.
