# Supplementary material for: The Buffer Effect of Social Identity on Psychological Stress in Different Competition Conditions
Source: Behav Sci (Basel). 2026 Mar 2;16(3):352. doi: 10.3390/bs16030352 (PMC13023628; doi:10.3390/bs16030352)
Supplement: Supplementary file 1 [file behavsci-16-00352-s001.zip › behavsci-4075622-supplementary.pdf]

Supplementary Materials for

The buffer-effect of social identity on psychological stress in different competition conditions

This document includes:

Table S1: STAI and baseline level of identity data in Experiment 1;

Table S2: Psychogenic Stress Validity Test in Experiment 1;

Table S3: Social identity highlights validity tests in Experiment 1;

Table S4: STAI and baseline level of identity data in Experiment 2;

Table S5: Psychogenic Stress Validity Test in Experiment 2;

Table S6: Social identity highlights validity tests in Experiment2;

Table S1: STAI and baseline level of identity data in Experiment 1

	Social identity<br>*M*±*SD*	Personal identity<br>*M*±*SD*	*t*
State Anxiety	41.22±8.26	39.64±8.10	.55
Trait anxiety	45.70±6.32	44.48±8.11	.42
Baseline level of identification	6.56±1.18	6.86±1.04	-1.01

Table S2: Psychogenic stress validity test in Experiment 1

	Baseline period *M*±*SD*	Mandate period *M*±*SD*	*F*	η_p^2
Subjective stress report	3.33±1.42	4.73±1.26	38.82^{***}	0.324
Heart rate	79.07±8.28	89.80±12.37	108.507^{***}	0.656
Systolic blood pressure	97.55±11.33	104.79±12.91	66.30^{***}	0.538
Diastolic blood pressure	57.65±9.00	65.13±9.12	19.646^{***}	0.556

Note: $*p < 0.05$, $**p < 0.01$, $***p < 0.001$

Table S3: Social identity highlights validity tests in Experiment 1

	Personal identity	Social identity	*F*	η_p^2
Identity operation score (*M*±*SD*)	4.68±0.88	5.22±0.69	9.98^{**}	0.154

Note: $*p < 0.05$, $**p < 0.01$, $***p < 0.001$

Table S4: STAI and baseline level of identity data in Experiment 2

Identity	Competition	*N*	Trait anxiety *M*±*SD*	State Anxiety *M*±*SD*	Baseline level of social identity *M*±*SD*
	Intragroup	30	44.40±6.89	40.80±8.93	6.90±1.04
Personal identit	Intergroup	30	42.23±5.12	38.63±6.71	7.09±1.07
	Total	60	43.32±6.11	39.71±7.91	6.99±1.05
	Intragroup	29	47.66±7.66	40.57±8.67	6.74±1.29
Social identity	Intergroup	30	43.67±8.30	41.56±9.33	7.10±0.99
	Total	59	45.63±8.18	41.08±8.95	6.92±1.15
Total	Intragroup	59	46.00±7.40	40.69±8.73	6.82±1.16
	Intergroup	60	42.95±6.88	40.10±8.19	7.09±1.02
	Total	119	44.46±7.27	40.39±8.43	6.96±1.10

Table S5: Psychogenic stress validity test in Experiment 2

	Baseline period *M*±*SD*	Mandate period *M*±*SD*	*F*	η_p^2
Subjective stress report	3.30± 1.44	4.75±1.25	127.16***	0.529
Heart rate	80.24±10.48	92.33±13.69	249.55***	0.673
Systolic blood pressure	98.66±8.97	106.91±9.91	153.98***	0.562
Diastolic blood pressure	58.06±9.49	65.19±9.41	112.75***	0.484

Note: *$p < 0.05$,**$p < 0.01$,***$p < 0.001$

Table S6: Social identity highlights validity tests in Experiment2

Independent variable	Categories	*N*	Dependent variable	*M*±*SD*	*F*	η_p^2
Competition	Intergroup	60	Competition Operations Score	6.30±2.17	7.86**	.062
	Intragroup	59		5.08±2.58		
Identity	Personal identit	60	Identity Operations Score	4.77±1.02	13.72**	.104
	Social identity	59		5.35±0.82		

Note: *$p < 0.05$,**$p < 0.01$,***$p < 0.001$
